# Preemptive Mpox Vaccine Deployment: Aligning Strategy With Reality

**DOI:** 10.1093/infdis/jiaf365

**Published:** 2025-07-21

**Authors:** Sung-mok Jung, Fuminari Miura, Hiroaki Murayama, Sebastian Funk, Jacco Wallinga, Justin Lessler, Akira Endo

**Affiliations:** Carolina Population Center, University of North Carolina at Chapel Hill; Centre for Infectious Disease Control, National Institute for Public Health and the Environment, Bilthoven, the Netherlands; Center for Marine Environmental Studies, Ehime University; School of Medicine, International University of Health and Welfare, Narita, Japan; Department of Infectious Disease Epidemiology; The Centre for Mathematical Modelling of Infectious Diseases, London School of Hygiene & Tropical Medicine, United Kingdom; Centre for Infectious Disease Control, National Institute for Public Health and the Environment, Bilthoven, the Netherlands; Department of Biomedical Data Sciences, Leiden University Medical Center, the Netherlands; Carolina Population Center, University of North Carolina at Chapel Hill; Department of Epidemiology, University of North Carolina at Chapel Hill; Department of Epidemiology, Johns Hopkins Bloomberg School of Public Health, Baltimore, Maryland; Saw Swee Hock School of Public Health, National University of Singapore; School of Tropical Medicine and Global Health, Nagasaki University, Japan

To the  Editor—We appreciate Tsagkaris and colleagues' commentary on our study [1], which draws attention to key aspects of mpox vaccine deployment in Greece and its broader relevance in the global context. We fully agree that logistical coordination, resilient infrastructure, and public trust are essential to an effective and equitable mpox immunization strategy, particularly under the pressure of sustained transmission as in Greece. Yet, beyond these broadly applicable challenges to vaccination efforts in outbreak settings, the mpox response highlights another fundamental but often underrecognized determinant of impact: the timing of vaccine deployment.

The 2022 global outbreak of clade IIb mpox illustrated how rapidly infection can saturate within highly heterogeneous sexual networks among men who have sex with men, once infection-acquired immunity accumulates among individuals with the highest numbers of sexual partners (so-called core groups [2]). These individuals are at the highest risk of infection and thus likely to be involved in the rapid spread in the earliest phase, but they are also the earliest to develop infection-derived immunity such that they no longer contribute to the epidemic progression in the later phase [3–5]. This dynamics, arising from the highly heterogeneous sexual network structure relevant to clade IIb mpox, creates a narrow time window in which vaccination can effectively interrupt onward transmission. However, in practice, vaccine deployment has been largely reactive, often initiated only after sustained local transmission has already taken hold, reflecting the inherent logistical and operational constraints in outbreak responses [6]. This delay is compounded by structural barriers, such as social stigma and limited diagnostic capacity, which impede timely case detection and compress the window for effective immunization.

Indeed, intensive vaccination rollout targeting high-risk populations took place in affected countries during the clade IIb outbreak, with around 336 000 doses administered in 25 European Union/European Economic Area countries between September 2022 and February 2023 [7]. However, retrospective analyses suggest that transmission might have already been on the decline before the majority of these doses were delivered [8]. While vaccines likely continued to mitigate individual disease burden—with an estimated 66%–85% effectiveness against disease [9]—the delay in deployment might have limited their population-level impact, particularly the potential indirect protection through herd immunity.

These constraints have prompted growing interest in more proactive strategies, with preemptive vaccination often discussed as a potential strategy [10]. However, translating this concept into practice presents several challenges. The limited pace of vaccine rollout in practice implies that immunization may need to begin even before the first importation is identified. Detecting a clear and actionable early signal that warrants such action remains inherently difficult, particularly when initial transmission is sporadic or underreported. Further complicating matters, initiating a preemptive vaccine campaign in the absence of confirmed local transmission poses a political dilemma: decision makers must carefully balance the perceived risk of overreaction against the potential consequences of delayed response.

Addressing these challenges requires structural foresight and a sustained investment in global early-warning capacity. Surveillance systems informed by real-time importation risk modeling can facilitate more timely and strategic decision making around preemptive vaccine deployment. At the same time, such immunization strategies must be responsive to the diversity of mpox transmission across clades and regions [11]. for instance, ongoing clade I outbreaks in several African countries involve broader routes of transmission, including household and community transmission affecting wider populations, in contrast with the observed patterns in the global clade IIb outbreak. As such, the timing and target of vaccination efforts must be tailored to local epidemiologic and social contexts to achieve maximum impact.

Above all, the challenges of mpox vaccine deployment reach beyond national borders, demanding coordinated global action. Ensuring timely sharing of outbreak information and equitable vaccine access, particularly in resource-limited settings, is imperative. A globally coordinated response will be essential to align preemptive immunization strategies with reality and maximize their potential to mitigate disease burden.

## Notes

***Disclaimer.*** The funders had no role in study design, data collection and analysis, decision to publish, or preparation of the manuscript.

***Financial support.*** This work was supported by the Centers for Disease Control and Prevention (SHEPheRD 200-2016-91781 to S.-m. J. and J. L.); the Japan Society for the Promotion of Science (Grants-in-Aid KAKENHI 20J00793 and 21KK0107 to F. M.); the Ministry of Education, Culture, Sports, Science and Technology (Leading Academia in Marine and Environment Pollution Research to F. M.); the HORIZON EUROPE European Research Council (HORIZON 2023 ESCAPE 101095619 to F. M. and J. W.); foundation for the Fusion of Science and Technology (to H. M. and A. E.); the Japan Science and Technology Agency Precursory Research for Embryonic Science and Technology (JPMJPR22R3 to H. M. and A. E.); Wellcome Trust (210758 to S. F.); the Japan Society for the Promotion of Science (Overseas Research Fellowships to A. E.); the Japan Society for the Promotion of Science (Grants-in-Aid KAKENHI 22K17329 to A. E.); the National University of Singapore (Startup Grant A-0009841 to A. E.); and the Japan Agency for Medical Research and Development (JP223fa627004).
